# Soluble terminal complement complex blood levels are elevated in schizophrenia

**DOI:** 10.1007/s00406-023-01738-z

**Published:** 2024-01-19

**Authors:** Susa Savukoski, Marco Mannes, Lisa Wohlgemuth, Anke Schultze, Paul C. Guest, Gabriela Meyer-Lotz, Henrik Dobrowolny, Borna Relja, Markus Huber-Lang, Johann Steiner

**Affiliations:** 1 Institute of Clinical and Experimental Trauma Immunology, University Hospital Ulm, University of Ulm, Ulm, Germany; 2 Department of Psychiatry, University Hospital Magdeburg, University of Magdeburg, Magdeburg, Germany; 3 Laboratory of Translational Psychiatry, University of Magdeburg, Magdeburg, Germany; 4 Laboratory of Neuroproteomics, Department of Biochemistry and Tissue Biology, Institute of Biology, University of Campinas (UNICAMP), Campinas, Brazil; 5 Department of Trauma, Hand, Plastic and Reconstructive Surgery, Translational and Experimental Trauma Research, University Hospital Ulm, University of Ulm, Ulm, Germany; 6 Center for Behavioral Brain Sciences (CBBS), Magdeburg, Germany; 7 Center for Health and Medical Prevention (CHaMP), Magdeburg, Germany; 8 German Center for Mental Health (DZPG), Center for Intervention and Research on Adaptive and Maladaptive Brain Circuits, underlying Mental Health (C-I-R-C), Halle-Jena-Magdeburg, Magdeburg, Germany

**Keywords:** Complement system, sTCC, C5a, C4, Classical pathway, First-episode psychosis, Confounders

## Abstract

The role of the complement system in schizophrenia (Sz) is inconclusive due to heterogeneity of the disease and study designs. Here, we assessed the levels of complement activation products and functionality of the classical pathway in acutely ill unmedicated Sz patients at baseline and after 6 weeks of treatment versus matched controls. The study included analyses of the terminal complement complex (sTCC) and C5a in plasma from 96 patients and 96 controls by enzyme-linked immunosorbent assay. Sub-group analysis of serum was conducted for measurement of C4 component and activity of the classical pathway (28 and 24 cases per cohort, respectively). We found no differences in levels of C5a, C4 and classical pathway function in patients versus controls. Plasma sTCC was significantly higher in patients [486 (392–659) ng/mL, *n* = 96] compared to controls [389 (304–612) ng/mL, *n* = 96] (*p* = 0.027, δ = 0.185), but not associated with clinical symptom ratings or treatment. The differences in sTCC between Sz and controls were confirmed using an Aligned Rank Transformation model considering the covariates age and sex (*p* = 0.040). Additional analysis showed that sTCC was significantly associated with C-reactive protein (CRP; *p* = 0.006). These findings suggest that sTCC plays a role in Sz as a trait marker of non-specific chronic immune activation, as previously described for CRP. Future longitudinal analyses with more sampling time points from early recognition centres for psychoses may be helpful to better understand the temporal dynamics of innate immune system changes during psychosis development.

**Supplementary Information:**

The online version contains supplementary material available at 10.1007/s00406-023-01738-z.

## Introduction

Recently, we found evidence of significantly elevated neutrophils in a subgroup of unmedicated schizophrenia (Sz) patients [19]. Symptom improvement following treatment was associated with decreased neutrophil counts, particularly in olanzapine-treated first-episode patients (FESz). Notably, only patients without clinical signs of infection were included in the study. This finding of innate immune system activation is consistent with evidence of elevated pro-inflammatory cytokines in blood [7] and cerebrospinal fluid (CSF) [21], and increased susceptibility to infections and autoimmunity in some patients [1].

**Fig. 1:**
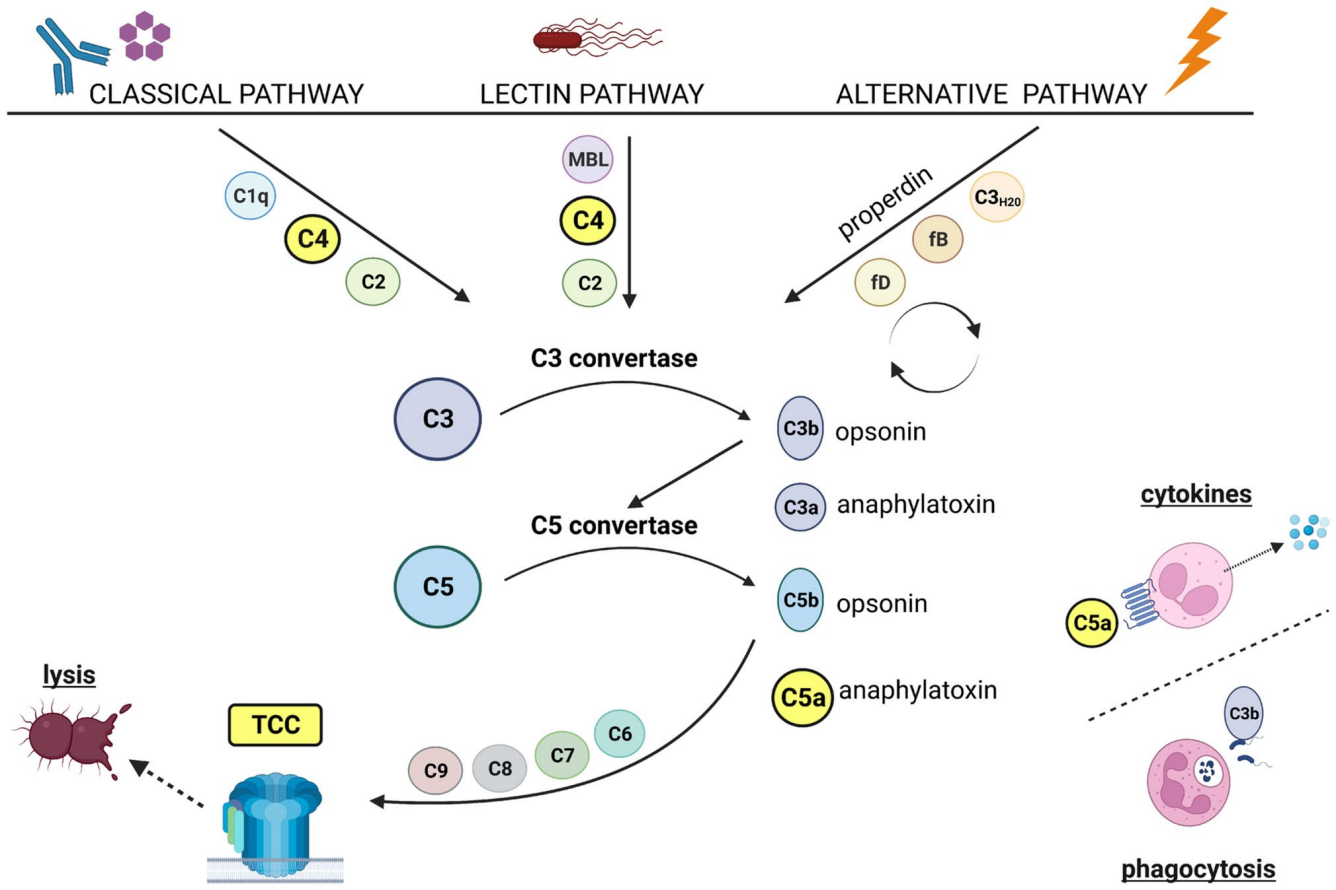
Illustration of the common complement activation pathways. The *classical pathway* is mainly triggered by antibody-antigen complexes or C-reactive protein, which are responsible for the further cleavage of the initial components C2 and C4 and the formation of C3 and C5 convertases. The functionally similar *lectin pathway* is activated by mannose-binding lectins (MBLs) and ficolins, which recognise sugar residues on pathogen surfaces and cause enzymatic cleavage of the initial complement components. The *alternative pathway* is spontaneously activated at low levels and is induced, for example, by artificial surfaces, leading to an amplification loop and the formation of a specific C3 convertase. All three pathways are converging into a *common terminal pathway* which causes lysis of the target cell. This is initiated by the cleaved C5 molecule, C5b, causing the subsequent binding of C6, C7, C8 and multiple C9 molecules in the form of a membrane-spanning pore or soluble activation product. There are also pathway-specific by-products, e.g. C4b/d, which bind to the surface of target cells, or general soluble fragments, e.g. C3a and C5a, which mediate their function by binding to their respective receptors. Lower right corner of the drawing: C3b can trigger phagocytosis, whilst C5a can induce, for example, the production of pro-inflammatory cytokines in neutrophils. Figure generated with Biorender (www.biorender.com)

As part of the immune response, the complement cascade consists of more than 30 proteins and can be initiated via the classical, lectin or alternative pathways (Fig. 1) [13]. The pathway is activated by a protease cascade generating soluble (e.g. C3a, C5a) and membrane-associated (e.g. C4b/d, C3b/iC3b) products. Clearance of pathogens and tissues is accomplished via assembly of the terminal complement complex (TCC, also known as C5b-9 or MAC), which mediates lytic and pro-inflammatory processes. However, in some diseases or traumas, complement dysfunction can lead to disrupted immune balance, which has been suggested to play a role in Sz [11].

In addition to these immune functions, the complement system orchestrates synaptic pruning during normal brain development and maturation via microglial cells [14]. It has therefore become a focus of Sz research in recent years, particularly after the discovery of complex genetic variation in MHC I and increased C4 expression in human post-mortem brains of Sz patients [15, 18].

To further understand the involvement of the peripheral complement system in Sz, our study provides comprehensive measurements of peripheral complement components in acutely ill unmedicated patients and matched healthy controls. Additionally, the functionality of the classical complement pathway was assessed. Our longitudinal study design with follow-up tests after 6 weeks of therapy and detailed clinical characterisation of patients, allowed consideration of the influence of disease stage, antipsychotic medication, smoking and other demographic variables on complement system effects.

## Methods

### Patients and controls

The study complied with German laws, the Declaration of Helsinki and institutional review guidelines. Participants gave written informed consent. Specimens were collected from sequentially admitted acutely ill Sz patients at the Department of Psychiatry, University of Magdeburg, Germany from May 2011 to August 2021 (*n* = 192) (Table S1) [19]. Patients were diagnosed following ICD10 [3] and AWMF-S3 guidelines [5]. FESz patients (*n* = 61) were antipsychotic-naïve at baseline (T0). Relapsed (RSz) patients (*n* = 35) were unmedicated ≥ 6 weeks before blood sampling. Control samples (*n* = 96) were from healthy donors, hospital staff and relatives over the same dates. Exclusion criteria included substance abuse, psychosis induced by medical conditions, immunotherapy, and infection. Controls were screened for personal/family history of neuropsychiatric disorders using the Mini-International Neuropsychiatric Interview [16]. Blood analyses and Positive and Negative Syndrome Scale (PANSS) assessments were performed at baseline. Six-week (T6) follow-up assessments were available for 57 patients. Types and cumulative dosages of antipsychotics (T0–T6) were converted to chlorpromazine (CPZ) equivalents and comprised olanzapine (*n* = 26), risperidone (*n* = 23), aripiprazole (*n* = 5), quetiapine (*n* = 2) and ziprasidone (*n* = 1).

### Samples and analyses

Blood was collected from fasting subjects at 08:00 AM into Vacutainer tubes (Becton Dickinson). EDTA-tubes were used to determine white blood cell (WBC) counts within 1 h. For plasma, additional EDTA-tubes were centrifuged immediately at 1000 g for 10 min. Serum tubes were centrifuged at 1000 g for 10 min after 2 h clotting. Plasma/serum supernatants were stored at  – 80 °C.

### Complement components

Plasma was used for C5a, sTCC and neutrophil priming analyses, and serum for C4 and haemolysis assays. WBC counts and C-reactive protein (CRP) were determined as previously described [19]. Complement pathway activation was measured by enzyme-linked immunosorbent assay (ELISA) for C5a (DRG Instruments GmbH) and sTCC (sC5b-9; OptEIA™ Set, Biosciences) (Table 1). C4 cleavage was determined in patients (*n* = 28) and controls (*n* = 28) also by ELISA (Abcam) (Table S2).

**Table 1:**
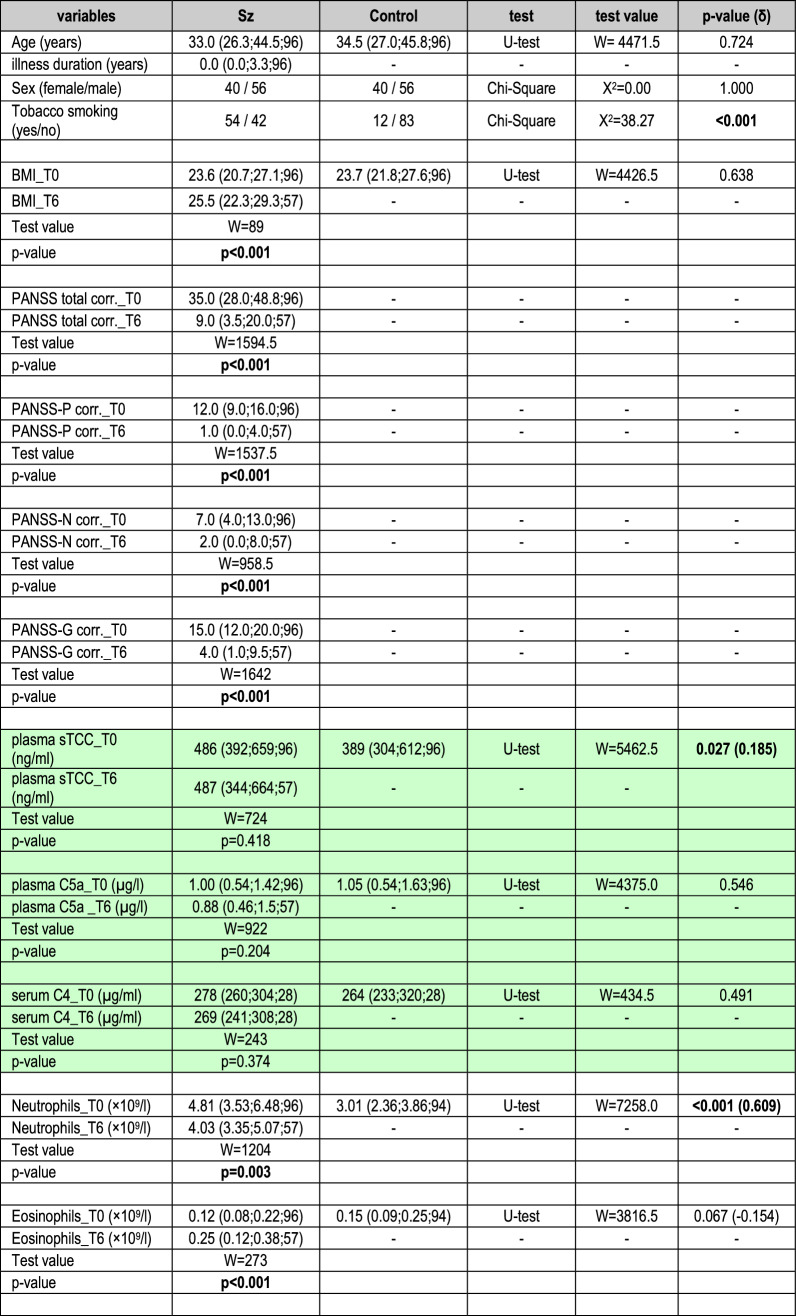

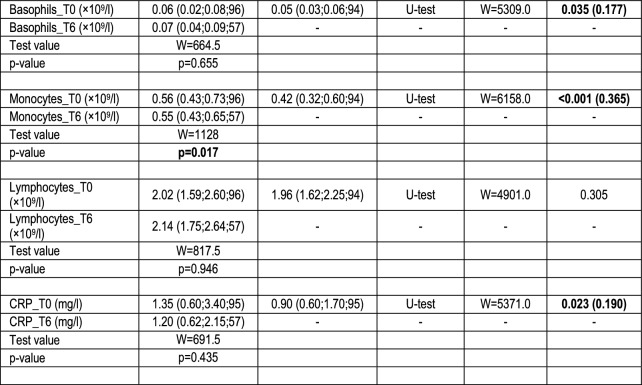
Comparison of Sz patients with controls

Demographic data, clinical assessments, complement measurements (sTCC, C5a and C4), WBC counts and CRP

Data are presented as median (quartile 1; quartile 3; sample size) or number of cases. Significant p values are highlighted in bold font. *CRP*  C-reactive protein, *δ*  effect size Cliff’s delta, *WBC*  white blood cell, *PANSS*  Positive and Negative Syndrome scale. PANSS scores were corrected (corr.) by subtraction of minimum scores, which represented no symptoms; T0 = baseline; T6 = follow-up after 6 weeks of treatment

### Haemolytic analysis

Random samples (Table S3) were measured for haemolysis of sheep red blood cells (shRBCs; Fiebig Animal Products). RBCs were washed with 5 mM PBS/EDTA, followed by exposure to 1:4000-diluted rabbit anti-sheep serum (Colorado Serum Company). Afterwards, 1% human serum was added and lysis measured by detection of released haemoglobin at 405 nm in a spectrophotometer. Water was used as a positive control (100% lysis) and 20 mM Mg–EGTA as a negative (0% lysis) control.

### PMN complement receptor expression

Polymorphonuclear cells (PMNs) were isolated by Ficoll-Paque (GE Healthcare) density gradient centrifugation, followed by dextran sedimentation. Erythrocytes were lysed and neutrophil-enriched granulocytes adjusted to 3 × 10^7^ cells/mL in Hank’s balanced salt solution containing calcium and magnesium (HBSS–Ca–Mg). Isolated PMNs from three healthy blood donors (both sexes, aged 21–40 years) were exposed to 3% plasma from 9 patients and 10 controls (3–4 plasma samples/PMN sample) for 10 min (Table S4) and stained with 150 ng/mL FITC-labelled CD11b, 68 ng/mL Pe-Cy7-labelled C3aR, and 135 ng/mL APC-labelled C5aR1 antibodies (BioLegend) for 15 min at 37 °C. Cells were fixed with 1% paraformaldehyde (pH 8.0) for 15 min at room temperature in darkness. Samples were centrifuged at 340 g for 5 min, the pellets resuspended in HBSS–Ca–Mg, and measured rapidly by cytometry (BD Biosciences). The flow rate was kept low, whilst 7500–10,000 events were measured.

### Statistics

Data analyses used R (v4.3.1). Group differences in sex and tobacco smoking were calculated using chi-squared tests. Corrected PANSS scores were derived by subtraction of minimum (no symptoms) from raw scores. As most data were not normally distributed (Shapiro–Wilk testing), group differences were calculated using non-parametric Mann–Whitney *U* tests. Cliff’s delta (δ) was used to assess effect sizes. Probatory analyses of covariance using Aligned Rank Transformation (ART) with stepwise inclusion of age, sex, BMI and number of cigarettes smoked/day as covariates were used to identify significant confounders. Relevant confounding factors were included as covariates in the final ART model to confirm diagnosis-dependent differences. Separate ART analyses with the covariates CRP or neutrophil count were calculated to determine if these inflammatory parameters were associated with sTCC, C5a and C4 measures. For significantly identified covariates, Spearman correlations were calculated in order to identify the direction of the relationships. A mixed-effects linear regression model was applied to analyse plasma priming data. The effect of different blood donors was accounted for using a random effects model to minimise variation in donor granulocyte responses, setting the baseline equal. Given the exploratory nature of this study, correction for multiple comparisons was not applied. Statistical tests were two-tailed with *p* < 0.05 considered significant.

## Results

### Demographic data and clinical assessments

Sz patients and controls were matched for age, sex and BMI (Table 1; Table S1). The proportion of smokers was higher in patients than controls and FESz had shorter illness durations than RSz patients. Baseline PANSS scores showed similar symptom severity in FESz and RSz patients. PANSS scores in all Sz patients and FESz and RSz subgroups improved significantly after 6 weeks of treatment.

### Complement system, WBC count and CRP analyses

#### Baseline

sTCC levels were significantly higher in patients [486 (392–659) ng/mL, *n* = 96] compared to controls [389 (304–612) ng/mL, *n* = 96] (δ = 0.185), but no significant differences were observed for C5a and C4 (Table 1). We also found significantly higher neutrophil (δ = 0.609) and monocyte (δ = 0.365) counts, and elevated CRP levels (δ = 0.190) in Sz compared to controls, consistent with our previous study [19]. Additionally, eosinophil counts were lower (δ =  – 0.154) and basophil counts higher (δ = 0.177) in Sz versus controls. FESz and RSz patients did not differ in any of these measures (Table S1).

### Follow-up

Levels of sTCC, C5a and C4 in patients did not change significantly after 6 weeks of treatment (Table 1). However, a significant decline in neutrophil and monocyte counts was observed in Sz whilst eosinophil counts increased. No significant changes were observed regarding basophils, lymphocytes and CRP. The FESz and RSz subgroups did not differ in these measures at week 6 (Table S1).

### Consideration of potential confounding factors

ART analyses with stepwise inclusion of age, sex, BMI and smoking as single covariates, identified age and sex as relevant confounders for sTCC, whilst BMI had a significant influence on C4 (Table 2). Further analyses of these confounders showed that sTCC blood levels correlated with age (ρ = 0.166, *p* = 0.021), and were higher in females versus males (*p* < 0.001). BMI showed a borderline significant correlation with C4 (ρ = 0.343, *p* = 0.050). Differences of sTCC between Sz and controls were confirmed by the final ART model using the covariates age and BMI (*p* = 0.040). Calculation of group statistics for C4 by ART with the covariate BMI still showed no significant difference between Sz patients and controls (*p* = 0.552). Notably, CRP levels were significantly confounded by BMI and smoking. When these covariates were taken into account, the diagnosis-related differences in CRP disappeared (*p* = 0.429).

**Table 2:**
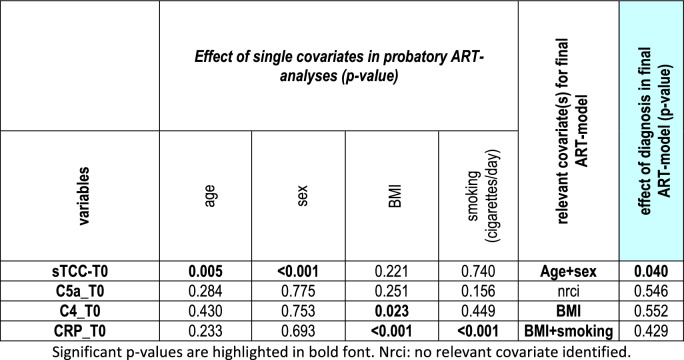
Covariate effect of age, sex, BMI, and smoking by probatory ART analyses and final ART model

Factors that had a significant influence on respective complement components or CRP were included as covariates in the final ART model (highlighted in blue)

Significant *p* values are highlighted in bold font. Nrci: no relevant covariate identified

### Consideration of possible complement factor/CRP or neutrophil associations

Separate ART analyses showed significant associations of CRP with sTCC and C5a (*p* = 0.006 each) and of neutrophil counts with C4 (*p* = 0.032; Table 3). Spearman correlations suggested that this was due to positive correlations of CRP with sTCC (ρ = 0.303, *p* < 0.001) and C5a (ρ = 0.196, *p* = 0.007), and of neutrophil count with C4 (ρ = 0.343, *p* = 0.010).

**Table 3:** Covariate effect of CRP and neutrophil count by probatory ART analyses

Variables	Effect of covariate CRP in ART analysis (p-value)	Effect of covariate neutrophil count in ART analysis (p-value)
sTCC-T0	**0.006**	0.492
C5a_T0	**0.006**	0.285
C4_T0	0.206	**0.032**

Samples were analysed using ART to identify significant physiological relationships between levels of sTCC, C5a and C4 with CRP or neutrophil counts in the same samples

Significant *p* values are highlighted in bold font

### Functional analyses

#### Haemolysis of shRBCs

No significant difference in haemolytic capacity was observed between patient (*n* = 24) and control (*n* = 24) sera at baseline (Table S2). Additionally, no change in activity was observed in the same samples between baseline and follow-up, and no differences were detected for FESz and RSz subgroups at either time-point.

### PMN complement receptor expression

Exploratory investigation of patient (*n* = 9) and control (*n* = 10) plasma priming effects on healthy granulocytes revealed no change in expression of complement receptors C5aR1, C3aR nor CD11b (Table S3). The same applied when the potential plasma priming effect was compared between baseline (*n* = 9) and follow-up (*n* = 9).

## Discussion

These findings suggest that sTCC plays a role in Sz, not as a state marker of acute psychosis, but rather as a trait marker of non-specific chronic immune activation. This is consistent with previous findings regarding elevated CRP levels in Sz regardless of antipsychotic use and disease stage [4, 19]. In addition, our current complementary statistical analysis showed that sTCC was significantly linked to CRP (*p* = 0.006). This subsequent activation of the complement cascade is a potent activator of neutrophils [9]. Indeed, we found an association of blood neutrophil counts with C4 (but not with sTCC and C5a).

CRP is a potential trigger of the classical pathway of the complement system (see top left of Fig. 1). Our study showed that CRP levels were significantly associated with BMI and smoking as previously described in the general population [2, 20]. The influence of these confounding factors on CRP was stronger than that of the diagnosis Sz. It should be noted that the observed changes in sTCC levels in Sz go beyond a pure relationship with CRP, as they were diagnosis-related in our final ART analysis, which controlled for relevant confounders.

Contrary to previous publications, we observed no significant correlations of complement factors with PANSS scores or WBC counts in Sz patients [12, 17]. However, we did find that sTCC correlated with age and C4 with BMI at baseline, consistent with previous studies [6, 10]. We also found that sTCC levels were higher in acutely ill Sz patients compared to controls with a small effect size and were significantly higher in female compared to male subjects. Furthermore, there were also significantly more smokers in the patient group than in the control group, but smoking had no significant association with any of the complement components.

There are limitations to this study. First, we measured complement levels in peripheral blood and not in CSF which may better reflect CNS effects and differ from circulating complement profiles [6]. Additionally, C4 levels and the functional studies were only assessed in a subset of subjects due to a low sample availability. Finally, we only measured total complement protein levels and not phosphorylation differences. This may be important as Jaros et al. found differences in phosphorylation state and not whole protein levels for several members of the complement cascade in Sz patients compared to controls [8].

## Conclusions and future perspectives

We found an increase in sTCC and elevated CRP levels in Sz patients compared to controls. However, there was no evidence of altered blood levels of C5a, C4 or peripheral classical complement pathway function in Sz patients versus controls. Also, none of the complement components were associated with severity of clinical symptoms and did not change after treatment. Taken together with the above findings, this indicates a potential influence of the complement cascade as a trait marker of non-specific chronic immune activation. Thus, we suggest further investigations including longitudinal analyses from early recognition centres. This may be helpful to improve our understanding of the temporal dynamics of innate immune system changes during psychosis development. Also, use of other measures of complement system activation should be considered, such as the application of targeted phosphoproteomic analyses.

### Supplementary Information

Below is the link to the electronic supplementary material.

Supplementary file1 (DOCX 32 KB)

## Data Availability

All data are either presented in the manuscript or supplementary materials. Additional information can be made available upon reasonable request.
