# Supplementary material for: Soluble terminal complement complex blood levels are elevated in schizophrenia
Source: Eur Arch Psychiatry Clin Neurosci. 2024 Jan 19;274(5):1215–22. doi: 10.1007/s00406-023-01738-z (PMC11226555; doi:10.1007/s00406-023-01738-z)
Supplement: Supplementary file 1 — Supplementary file1 (DOCX 32 KB) [file 406_2023_1738_MOESM1_ESM.docx]

**Soluble terminal complement complex blood levels are**

**elevated in schizophrenia**

Susa Savukoski^1^, Marco Mannes^1^, Lisa Wohlgemuth^1^, Anke Schultze^1^, Paul C. Guest^2,3,4^,

Gabriela Meyer-Lotz^2,3^, Henrik Dobrowolny^2,3^, Borna Relja^5^,

Markus Huber-Lang^1^, Johann Steiner^2,3,6,7,8^*

^1^Institute of Clinical and Experimental Trauma Immunology, University Hospital Ulm, University of Ulm, Ulm, Germany

^2^Department of Psychiatry, University Hospital Magdeburg, University of Magdeburg, Magdeburg, Germany

^3^Laboratory of Translational Psychiatry, University of Magdeburg, Magdeburg, Germany

^4^Laboratory of Neuroproteomics, Department of Biochemistry and Tissue Biology, Institute of Biology, University of Campinas (UNICAMP), Campinas, Brazil

^5^Department of Trauma, Hand, Plastic and Reconstructive Surgery, Translational and Experimental Trauma Research, University Hospital Ulm, University of Ulm, Ulm, Germany

^6^Center for Behavioral Brain Sciences (CBBS), Magdeburg, Germany

^7^Center for Health and Medical Prevention (CHaMP), Magdeburg, Germany

^8^German Center for Mental Health (DZPG), Center for Intervention and Research on Adaptive and Maladaptive Brain Circuits underlying Mental Health (C-I-R-C), Halle-Jena-Magdeburg, Germany

*Corresponding Author:

Johann Steiner

Department of Psychiatry, University of Magdeburg

Leipziger Str. 44, 39120 Magdeburg, Germany

johann.steiner@med.ovgu.de

orcid.org/0000-0002-2611-2268

**Table S1:** **Comparison of FESz and RSz patients.** Demographic data, clinical assessments, complement measures (sTCC, C5a and C4), WBC count and CRP.

| **variables** | **FESz** | **RSz** | **test** | **test value** | **p-value** |
| --- | --- | --- | --- | --- | --- |
| Age (years) | 31.0 (26.0;42.0;61) | 37.0 (27.0;48.0;35) | U-test | W=896.5 | 0.194 |
| Illness duration (years) | 0.0 (0.0;0.0;61) | 6.0 (2.0;10.0;33) | U-test | W=61.0 | **<0.001** |
| Sex (female/male) | 28 / 33 | 12 / 23 | Chi-Square | X^2^=0.80 | 0.370 |
| Tobacco smoking (yes/no) | 32 / 29 | 22 / 13 | Chi-Square | X^2^=0.60 | 0.438 |
|  |  |  |  |  |  |
| BMI_T0 | 23.7 (21.1;27.5;61) | 23.3 (20.3;26.8;35) | U-test | W=1150.5 | 0.530 |
| BMI_T6 | 25.6 (22.5;29.7;42) | 24.7 (21.5;27.8;15) | U-test | W=333.0 | 0.751 |
| Test value | W=63 | W=1 |  |  |  |
| p-value | **p<0.001** | **p<0.001** |  |  |  |
|  |  |  |  |  |  |
| PANSS total corr._T0 | 36.0 (29.0;50.0;61) | 31.0 (24.0;42.0;35) | U-test | W=1309.0 | 0.066 |
| PANSS total corr._T6 | 14.0 (3.8;23.3;42) | 5.0(3.0;9.0;15) | U-test | W=422.5 | 0.052 |
| Test value | W=860 | W=120 |  |  |  |
| p-value | **p<0.001** | **p<0.001** |  |  |  |
|  |  |  |  |  |  |
| PANSS-P corr._T0 | 13.0 (9.0;15.5;61) | 12.0 (9.0;18.0;35) | U-test | W=1051.5 | 0.906 |
| PANSS-P corr._T6 | 2.0 (0.0;5.0;42) | 0 (0;1;15) | U-test | W=430.5 | **0.030** |
| Test value | W=861 | W=104 |  |  |  |
| p-value | **p<0.001** | **p=0.001** |  |  |  |
|  |  |  |  |  |  |
| PANSS-N corr._T0 | 8.0 (4.0;14.5;61) | 6.0 (2.0;11.0;35) | U-test | W=1297.0 | 0.081 |
| PANSS-N corr._T6 | 4.0 (0.0;10.3;42) | 1.0 (0.0;5.0;15) | U-test | W=400.0 | 0.114 |
| Test value | W=557.5 | W=57 |  |  |  |
| p-value | **p<0.001** | **p=0.036** |  |  |  |
|  |  |  |  |  |  |
| PANSS-G corr._T0 | 16.0 (12.5;22.0;61) | 13.0 (11.0;19.0;35) | U-test | W=1350.5 | **0.031** |
| PANSS-G corr._T6 | 5.5 (1.0;11.0;42) | 3.0 (0.0;5.0;15) | U-test | W=415.5 | 0.068 |
| Test value | W=893 | W=120 |  |  |  |
| p-value | **p<0.001** | **p<0.001** |  |  |  |
|  |  |  |  |  |  |
| plasma sTCC_T0 (ng/ml) | 462 (390;651;61) | 519 (401;754;35) | U-test | W=920.0 | 0.263 |
| plasma sTCC_T6 (ng/ml) | 480 (329;632;42) | 514 (357;767;15) | U-test | W=292.5 | 0.690 |
| Test value | W=377 | W=58 |  |  |  |
| p-value | p=0.359 | p=0.934 |  |  |  |
|  |  |  |  |  |  |
| plasma C5a_T0 (µg/l) | 0.87 (0.49;1.42;61) | 1.10 (0.57;1.47;35) | U-test | W=947.0 | 0.361 |
| plasma C5a _T6 (µg/l) | 0.85 (0.39;1.46;42) | 0.95 (0.54;1.53;15) | U-test | W=262.0 | 0.341 |
| Test value | W=495 | W=69 |  |  |  |
| p-value | p=0.407 | p=0.315 |  |  |  |
|  |  |  |  |  |  |
| serum C4_T0 (µg/ml) | 276 (264;324;21) | 281 (220;301;7) | U-test | W=88.0 | 0.458 |
| serum C4_T6 (µg/ml) | 272 (261;325;21) | 235 (232;276;7) | U-test | W=111.0 | **0.048** |
| Test value | W=128 | W=22 |  |  |  |
| p-value | p=0.683 | p=0.219 |  |  |  |
|  |  |  |  |  |  |
| Neutrophils_T0 (×10^9^/l) | 5.08 (3.59;6.58;61) | 4.51 (3.46;6.22;35) | U-test | W=1176.0 | 0.411 |
| Neutrophils_T6 (×10^9^/l) | 4.02 (3.31;5.21;42) | 4.03 (3.45;4.97;15) | U-test | W=323.0 | 0.893 |
| Test value | W=625 | W=99 |  |  |  |
| p-value | **p=0.029** | **p=0.026** |  |  |  |
|  |  |  |  |  |  |
| Eosinophils_T0 (×10^9^/l) | 0.10 (0.08;0.20;61) | 0.14 (0.08;0.24;35) | U-test | W=873.0 | 0.140 |
| Eosinophils_T6 (×10^9^/l) | 0.22 (0.12;0.38;42) | 0.28 (0.12;0.44;15) | U-test | W=289.5 | 0.650 |
| Test value | W=162 | W=15 |  |  |  |
| p-value | **p<0.001** | **p=0.008** |  |  |  |
|  |  |  |  |  |  |
| Basophils_T0 (×10^9^/l) | 0.06 (0.01;0.09;61) | 0.06 (0.04;0.08;35) | U-test | W=1138.5 | 0.589 |
| Basophils_T6 (×10^9^/l) | 0.06 (0.03;0.08;42) | 0.07 (0.05;0.09;15) | U-test | W=246.5 | 0.216 |
| Test value | W=395 | W=35 |  |  |  |
| p-value | p=0.728 | p=0.169 |  |  |  |
|  |  |  |  |  |  |
| Monocytes_T0 (×10^9^/l) | 0.60 (0.44;0.76;61) | 0.55 (0.41;0.66;35) | U-test | W=1250.5 | 0.165 |
| Monocytes_T6 (×10^9^/l) | 0.56 (0.42;0.70;42) | 0.50 (0.43;0.58;15) | U-test | W=382.0 | 0.228 |
| Test value | W=611 | W=84 |  |  |  |
| p-value | **p=0.047** | p=0.188 |  |  |  |
|  |  |  |  |  |  |
| Lymphocytes_T0 (×10^9^/l) | 2.03 (1.53;2.58;61) | 1.98 (1.70;2.64;35) | U-test | W=1012.0 | 0.675 |
| Lymphocytes_T6 (×10^9^/l) | 2.14 (1.76;2.63;42) | 2.15 (1.46;2.67;15) | U-test | W=327.5 | 0.828 |
| Test value | W=448.5 | W=63 |  |  |  |
| p-value | p=0.975 | p=0.890 |  |  |  |
|  |  |  |  |  |  |
| CRP_T0 (mg/l) | 1.26 (0.60;3.60;60) | 1.40 (0.60;3.30;35) | U-test | W=976.5 | 0.571 |
| CRP_T6 (mg/l) | 1.28 (0.68;2.20;42) | 1 (1;2;15) | U-test | W=351.0 | 0.519 |
| Test value | W=393 | W=48 |  |  |  |
| p-value | p=0.350 | p=0.889 |  |  |  |
|  |  |  |  |  |  |

Data are presented as median (quartile 1; quartile 3; sample size) or number of cases. Significant p-values are highlighted in bold font. CRP= C-reactive protein; WBC= white blood cell; PANSS= Positive and Negative Syndrome scale. PANSS scores were corrected (corr.) by subtraction of minimum scores, which represented no symptoms; T0= baseline; T6= follow-up after 6 weeks treatment.

**Table S2:** **Subgroup of subjects for the hemolysis experiments in shRBCs.** Demographic data, clinical assessments, hemolysis shRBCs exposed to 1% human serum, WBC count and CRP.

| **variables** | **Sz** | **Control** | **test** | **test value** | **p-value** |
| --- | --- | --- | --- | --- | --- |
| Age (years) | 30.5 (27.0;49.3;24) | 31.5 (25.0;48.0;24) | U-test | W = 308.5 | 0.680 |
| illness duration (years) | 0 (0;0;24) | - | - | - | - |
| Sex (female/male) | 7 / 17 | 6 / 18 | Chi-Square | X^2^=0.00 | 1.000 |
| Tobacco smoking (yes/no) | 13 / 11 | 2 / 22 | Chi-Square | X^2^= 9.70 | **0.002** |
|  |  |  |  |  |  |
| BMI_T0 | 24.5 (22.1;28.5;24) | 23.1 (21.9;25.1;24) | U-test | W = 334.5 | 0.343 |
| BMI_T6 | 25.7 (23.2;28.9;24) | - | - | - | - |
| Test value | W=23 |  |  |  |  |
| p-value | **p<0.001** |  |  |  |  |
|  |  |  |  |  |  |
| PANSS total corr._T0 | 35.5 (29.0;39.8;24) | - | - | - | - |
| PANSS total corr._T6 | 9.0 (3.3;21.5;24) | - | - | - | - |
| Test value | W=276 |  |  |  |  |
| p-value | **p<0.001** |  |  |  |  |
|  |  |  |  |  |  |
| PANSS-P corr._T0 | 11.0 (8.0;14.0;24) | - | - | - | - |
| PANSS-P corr._T6 | 1.5 (0.0;4.0;24) | - | - | - | - |
| Test value | W=300 |  |  |  |  |
| p-value | **p<0.001** |  |  |  |  |
|  |  |  |  |  |  |
| PANSS-N corr._T0 | 7.5 (4.0;14.5;24) | - | - | - | - |
| PANSS-N corr._T6 | 4.5 (0.0;9.5;24) | - | - | - | - |
| Test value | W=214.5 |  |  |  |  |
| p-value | **p=0.004** |  |  |  |  |
|  |  |  |  |  |  |
| PANSS-G corr._T0 | 13.5 (12.0;17.5;24) | - | - | - | - |
| PANSS-G corr._T6 | 4.0 (0.25;7.8;24) | - | - | - | - |
| Test value | W=299 |  |  |  |  |
| p-value | **p<0.001** |  |  |  |  |
|  |  |  |  |  |  |
| shRBCs hemolysis_T0 (%) | 42.8 (28.8;83.2;24) | 48.6 (33.8;86.2;24) | U-test | W = 259.0 | 0.557 |
| shRBCs hemolysis_T6 (%) | 46.3 (32.0;86.3;24) | - | - | - |  |
| Test value | W=120 |  |  |  |  |
| p-value | p=0.406 |  |  |  |  |
|  |  |  |  |  |  |
| Neutrophils_T0 (×10^9^/l) | 5.77 (4.44;7.58;24) | 2.70 (2.11;4.21;23) | U-test | W = 483.0 | **<0.001** |
| Neutrophils_T6 (×10^9^/l) | 4.02(2.80;5.66;24) | - | - | - |  |
| Test value | W=239 |  |  |  |  |
| p-value | **p=0.010** |  |  |  |  |
|  |  |  |  |  |  |
| Eosinophils_T0 (×10^9^/l) | 0.15 (0.10;0.26;24) | 0.16 (0.09;0.35;23) | U-test | W = 266.0 | 0.840 |
| Eosinophils_T6 (×10^9^/l) | 0.30 (0.19;0.38;24) | - | - | - |  |
| Test value | W=34 |  |  |  |  |
| p-value | **p<0.001** |  |  |  |  |
|  |  |  |  |  |  |
| Basophils_T0 (×10^9^/l) | 0.08 (0.00;0.09;24) | 0.05 (0.02;0.07;23) | U-test | W = 325.5 | 0.293 |
| Basophils_T6 (×10^9^/l) | 0.07 (0.05;0.10;24) | - | - | - |  |
| Test value | W=104 |  |  |  |  |
| p-value | p=0.194 |  |  |  |  |
|  |  |  |  |  |  |
| Monocytes_T0 (×10^9^/l) | 0.64 (0.53;0.76;24) | 0.42 (0.29;0.62;23) | U-Test | W = 450.5 | **<0.001** |
| Monocytes_T6 (×10^9^/l) | 0.58 (0.48;0.70;24) | - | - | - |  |
| Test value | W=223 |  |  |  |  |
| p-value | **p=0.038** |  |  |  |  |
|  |  |  |  |  |  |
| Lymphocytes_T0 (×10^9^/l) | 1.95 (1.46;2.61;24) | 1.94 (1.60;2.44;23) | U-Test | W = 283.0 | 0.890 |
| Lymphocytes_T6 (×10^9^/l) | 2.17 (1.84;2.68;24) | - | - | - |  |
| Test value | W=126 |  |  |  |  |
| p-value | p=0.509 |  |  |  |  |
|  |  |  |  |  |  |
| CRP_T0 (mg/l) | 1.21 (0.60;5.75;24) | 0.95 (0.60;1.67;24) | U-Test | W = 333.5 | 0.349 |
| CRP_T6 (mg/l) | 1.15 (0.70;2.30;24) | - | - | - |  |
| Test value | W=154 |  |  |  |  |
| p-value | p=0.187 |  |  |  |  |

Data are presented as median (quartile 1; quartile 3; sample size) or number of cases. Significant p-values are highlighted in bold font. CRP= C-reactive protein; WBC= white blood cell; PANSS= Positive and Negative Syndrome scale. PANSS scores were corrected (corr.) by subtraction of minimum scores, which represented no symptoms; T0= baseline; T6= follow-up after 6 weeks treatment.

**Table S3:** **Subgroup of subjects for the plasma priming effects on the expression of complement receptors on PMNs from healthy donors.** Demographic data, clinical assessments, expression of surface complement receptors C5aR1, C3aR, and CD11b on PMNs.

| **variables** | **Sz** | **Control** | **test** | **test value** | **p-value** |
| --- | --- | --- | --- | --- | --- |
| Age (years) | 29.0 (28.0;48.0;9) | 40.0 (30.5;49.3;10) | U-Test | W = 36.0 | 0.487 |
| illness duration (years) | 0 (0;2;9) | - | - | - | - |
| Sex (female/male) | 3 / 6 | 7 / 3 | Chi-Square | X^2^=1.30 | 0.255 |
| Tobacco smoking (yes/no) | 1 / 8 | 2 / 8 | Chi-Square | X^2^= 0.00 | 1.000 |
|  |  |  |  |  |  |
| BMI_T0 | 22.2 (19.9;27.5;9) | 25.0 (21.9;30.1;10) | U-Test | W = 31.0 | 0.278 |
| BMI_T6 | 24.2 (22.1;28.2;9) | - | - | - |  |
| Test value | W=0 |  |  |  |  |
| p-value | **p=0.004** |  |  |  |  |
|  |  |  |  |  |  |
| PANSS total corr._T0 | 35.0 (22.5;48.5;9) | - | - | - | - |
| PANSS total corr._T6 | 12.0 (4.0;21.0;9) | - | - | - | - |
| Test value | W=45 |  |  |  |  |
| p-value | **p=0.004** |  |  |  |  |
|  |  |  |  |  |  |
| PANSS-P corr._T0 | 13.0 (10.0;18.0;9) | - | - | - | - |
| PANSS-P corr._T6 | 4.0 (0.0;5.0;9) | - | - | - | - |
| Test value | W=44 |  |  |  |  |
| p-value | **p=0.013** |  |  |  |  |
|  |  |  |  |  |  |
| PANSS-N corr._T0 | 7.0 (2.5;10.5;9) | - | - | - | - |
| PANSS-N corr._T6 | 5.0 (1.0;9.0;9) | - | - | - | - |
| Test value | W=16 |  |  |  |  |
| p-value | p=0.799 |  |  |  |  |
|  |  |  |  |  |  |
| PANSS-G corr._T0 | 13.0 (11.5;19.5;9) | - | - | - | - |
| PANSS-G corr._T6 | 3.0 (0.0;6.5;9) | - | - | - | - |
| Test value | W=45 |  |  |  |  |
| p-value | **p=0.009** |  |  |  |  |
|  |  |  |  |  |  |
| C5aR1_T0 (MFI)* | 1571 (1273;2062;9) | 1394 (1070;1958;10) | U-Test | W = 54.0 | 0.497 |
| C5aR1_T6 (MFI)* | 1454 (1405;2234;9) | - | - | - |  |
| Test value | W=11 |  |  |  |  |
| p-value | p=0.203 |  |  |  |  |
|  |  |  |  |  |  |
| C3aR_T0 (MFI)* | 284 (269;312;9) | 282 (232;319;10) | U-Test | W = 49.5 | 0.744 |
| C3aR_T6 (MFI)* | 288 (256;297;9) | - | - | - |  |
| Test value | W=31.5 |  |  |  |  |
| p-value | p=0.314 |  |  |  |  |
|  |  |  |  |  |  |
| CD11b_T0 (MFI)* | 1984 (1480;2085;9) | 1697 (1578;1742;10) | U-Test | W = 56.5 | 0.369 |
| CD11b_T6 (MFI)* | 1855 (1531;2190;9) | - | - | - |  |
| Test value | W=23 |  |  |  |  |
| p-value | p=1.000 |  |  |  |  |
|  |  |  |  |  |  |

Data are presented as median (quartile 1; quartile 3; sample size) or number of cases. PANSS= Positive and Negative Syndrome scale. PANSS scores were corrected (corr.) by subtraction of minimum scores, which represented no symptoms; T0= baseline; T6= follow-up after 6 weeks treatment.

* The expression of C5aR1, C3aR and CD11b was assessed by flow cytometry. Median fluorescent intensities (MFI) are shown for APC, PE-Cy7, and FITC, respectively.
